# Inhibitory Effect of a Novel Antirheumatic Drug T-614 on the IL-6-Induced RANKL/OPG, IL-17, and MMP-3 Expression in Synovial Fibroblasts from Rheumatoid Arthritis Patients

**DOI:** 10.1155/2015/214683

**Published:** 2015-07-27

**Authors:** Yu Wei, Xiaoxun Sun, Minhui Hua, Wenfeng Tan, Fang Wang, Miaojia Zhang

**Affiliations:** Department of Rheumatology, The First Affiliated Hospital of Nanjing Medical University, 300 Guangzhou Road, Nanjing 210029, China

## Abstract

T-614 (also named as iguratimod), a novel antirheumatic drug, could attenuate joint inflammation and articular damage in rheumatoid arthritis (RA) patients, providing a new therapy for RA. Here, we tested the role T-614 on the IL-6-induced receptor activator of nuclear factor *κ*B ligand (RANKL)/osteoprotegerin (OPG), IL-17, and MMP-3 expression in synovial fibroblasts from rheumatoid arthritis (RASFs) patients. T-614 decreased RANKL expression and RANKL/OPG ratio in IL-6-induced RASFs. We confirmed this effect by a decrease of the mRNA and protein RANKL and mRNA RANKL/OPG in RASFs exposed in vitro to T-614 or MTX. Markedly decreased levels of IL-17, retinoid-related orphan receptor C (RORc), and MMP-3 mRNA expression were also observed in IL-6-induced RASFs in the presence of T-614 or MTX compared with those in its absence. Furthermore, T-614 blocked expression of p-ERK1/2 protein without affecting ERK1/2 expression, indicating that the way that T-614 regulated RANKL expression might be ERK1/2 pathway. Our results suggest that T-614 yields a strong improvement in arthritis via exact suppression of RANKL/OPG, IL-17, and MMP-3 expression in RASFs.

## 1. Introduction

Rheumatoid arthritis (RA) is a complicated autoimmune disease characterized by persistent synovitis and, thereby, bone erosion. Although the etiology is not fully understood, a combination of genetic and environmental risk factors contributes to breaching of the immune tolerance, playing an important role in RA pathogenesis. The goal of RA treatment is remission or low disease activity, ultimately slowing or preventing the progression of joint destruction.

T-614 (also named iguratimod), a novel disease-modifying antirheumatic drug, has been widely used in clinics in China and Japan. T-614 could inhibit the production of various inflammatory cytokines, including interleukin-1, interleukin-6, interleukin-8, and TNF and dramatically improve symptoms in RA patients [1] and collagen-induced arthritis (CIA) mice [2]. Moreover, T-614 could markedly suppress disease progression and bone erosion in CIA mice [3]; however, the exact mechanism remains unclear.

Receptor activator of nuclear factor *κ*B ligand (RANKL) is the most essential factor for osteoclastogenesis by stimulation of osteoclast precursor cells differentiating into osteoclasts and by prompting osteoclasts migration, fusion, activation, and survival [4]. Accumulated evidences have suggested that RANKL level was associated with bone erosion in RA [5]. OPG acts as the natural inhibitor of RANKL, preventing RANKL from binding to its osteoclast receptor. OPG knock-out mice exhibit severe osteoporosis and bone erosion [6], implicating the importance of RANKL/OPG balance for maintaining osteoclast homeostasis.

Proinflammatory cytokines including TNF-*α*, IL-17, IL-1*β*, and IL-6, derived from synovial fibroblasts of inflamed joints, are the primary trigger for the local or systemic high expression of RANKL in mice and RA patients [7, 8], which is one of the main mechanisms of inflammation-induced bone loss in RA. In addition to RANKL, IL-17 could directly increase cartilage proteoglycan loss and prompt the expansion of osteoclast precursors, participating in joint degradation in RA [9]. MMP-3 is also involved in bone destruction in RA patients by providing space for RASFs to invade through removing the extracellular matrix [10]. Here, we study the effect of T-614 on the RANKL, OPG, IL-17, and MMP-3 expression in a simulative inflammatory context by RASFs stimulated with IL-6 and conducted a comparative analysis of the antiarthritic of T-614 and MTX, the classic disease-modifying antirheumatic drugs (DMARDs) used for RA therapy.

## 2. Materials and Methods

### 2.1. Cell Culture and Reagents

Synovial tissues were obtained from active RA patients who were undergoing surgery for knee replacement surgery. All RA patients fulfilled the American College of Rheumatology revised criteria for the diagnosis of RA [11]. Synovial tissues were harvested and incubated with collagenase type I (1 mg/mL) for 2 hours at 37°C. After digestion, RASFs were washed extensively and cultured in DMEM supplemented with 10% FCS in a humidified 5% CO_2_ atmosphere. After overnight culture, nonadherent cells were removed, and adherent cells were cultured in DMEM supplemented with 10% FCS. RASFs were used between passages 3 and 7.

### 2.2. T-614 and MTX (Methotrexate) Treatment

RASFs (1 ∗ 10^5^/mL) were cultured in six-well plates and stimulated with IL-6 (Peprotech, Rocky Hill, USA)/sIL-6R (GenWay, San Diego, CA, USA) for 72 h in the presence or absence of T-614 or MTX (4.5 *μ*g/mL), using RASFs without IL-6/sIL-6R treatment as control. Recombinant human IL-6 and sIL-6R were used at 20 ng/mL and 100 ng/mL, respectively. T-614 was synthesized at Simcere Pharmaceutical (Nanjing, China), and it was used as solution in dimethyl sulfoxide (DMSO). The final concentrations of DMSO were less than 0.1% where the cell viability was not affected.

### 2.3. Cell Viability Assay

RASFs were seeded at a density of 3–5 ∗ 10^3^/0.2 mL/well in 96-well flat bottom. Then, RASFs were stimulated in different concentrations of T-614 (0, 20, 50, 100, and 150 *μ*g/mL) for 72 h. Viability assay was performed using the Cell Counting Kit-8 (Dojindo, Tokyo, Japan) according to manufacturer's instruction. Substrate was added to elicit a colorimetric reaction, and absorbance was measured at 450 nm using a microplate reader.

### 2.4. Real-Time PCR

RASFs, cultured with cytokines, T-614, and MTX or not, were harvested and total cellular RNA was isolated from cells extracted with the TRIzol reagent (Invitrogen, USA). Precipitated RNA was reverse transcribed by the PrimeScript RT regent Kit (TaKaRa, Japan). mRNA expression of *β*-actin and the selected molecules were determined by real-time PCR using SYBR Green Master Mix (Applied Biosystems, Foster City, CA, USA). Real-time PCR was performed using specific primers for (*β*-actin, sense 5′-CCACACTGTGCCCATCTACG-3′ and antisense 5′-AGGATCTTCATGAGGTAGTCAGTCAG-3′; IL-17, sense 5′-GGGCCTGGCTTCTGTCTGA-3′ and antisense 5′-AAGTTCGTTCTGCCCCATCA-3′; RORc, sense 5′-GAAGTGGTGCTGGTTAGGATGTG-3′ and antisense 5′-GCCACCGTATTTGCCTTCAA-3′; RANKL, sense 5′-CAACATATCGTTGGATCACAGCA-3′ and antisense 5′-GACAGACTCACTTTATGGGAACC-3′; OPG, sense 5′-GCGCTCGTGTTTCTGGACA-3′ and antisense 5′-AGTATAGACACTCGTCACTGGTG-3′; MMP-3, sense 5′-CGGTTCCGCCTGTCTCAAG-3′ and antisense 5′-CGCCAAAAGTGCCTGTCTT-3′). Thermocycler conditions included an initial holding at 50°C for 2 min and then 95°C for 10 min; this was followed by a 2-step PCR program: 95°C for 15 s and 60°C for 60 s for 40 cycles. Data were collected and quantitatively analyzed on an ABI PRISM 7900 sequence detection system (Applied Biosystems, CA, USA). *β*-Actin gene was used as an endogenous control. The amount of gene expression was then calculated as the difference cycle threshold (ΔCT) between the CT value of the target gene and the *β*-actin. ΔΔCT were differences between ΔCT values of test sample and the control. Relative expression of target genes was calculated as 2^−ΔΔCT^.

### 2.5. Western Blot Analyses

Cells were treated with IL-6/sIL-6R for 0, 1, 6, 12, 24, and 48 hours. For another experiment, RASFs were cultured with IL-6/sIL-6R plus T-614 or MTX for 72 hours, using 25 *μ*M PD98059 (CST, USA) before IL-6/sIL-6R was added as control. Proteins were extracted in lysis buffer (30 mM Tris [pH 7.5], 150 mM sodium chloride, 1 mM PMSF, 1 mM sodium orthovanadate, 1% nonidet P-40, 10% glycerol, and phosphatase and protease inhibitors). The proteins were then separated by SDS-PAGE and electrophoretically transferred onto polyvinylidene fluoride membranes. The membranes were probed with RANKL (EPR4999, Abcam, Cambridge, MA, USA), p-ERK1/2 (D13.14.4E, Cell Signaling, MA, USA), ERK1/2 (137F5, Cell Signaling, MA, USA) or GAPDH (D16H11, Cell Signaling, MA, USA), or *β*-actin (13E5, Cell Signaling, MA, USA) overnight at 4°C and then incubated with an HRP-coupled secondary Ab (Cell Signaling, MA, USA). Proteins were detected with the SuperSignal West Pico Chemiluminescent Kit (Thermo Scientific, Rockford, IL, USA). Densitometry values were analyzed and quantified with ImageLab (Bio-Rad).

### 2.6. Statistical Analysis

Data are presented as mean ± SD. Statistical analysis was performed using ANOVA test. A probability < 0.05 was defined as significant.

## 3. Results

### 3.1. RASFs Cultured In Vitro

**Figure 1:**
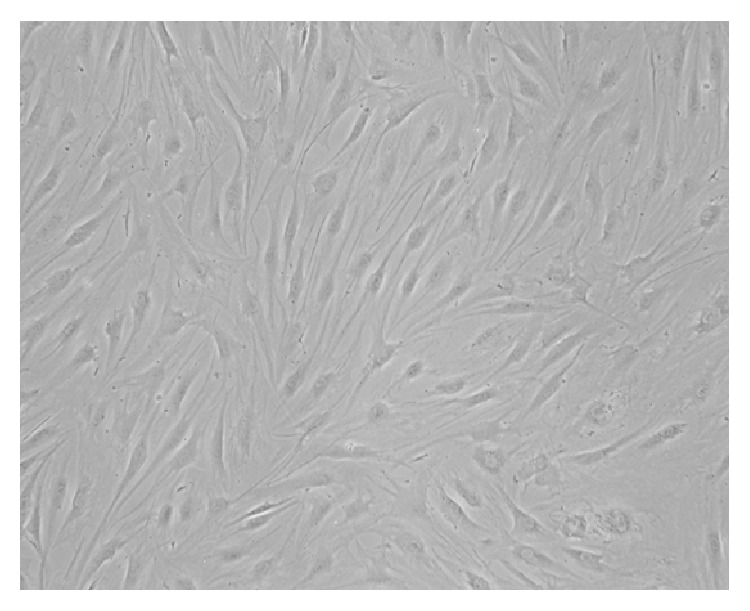
Appearance of human rheumatoid arthritis synovial fibroblasts at generation 4.

A homogeneous population (<1% CD11b, <2.5% CD14+, <1%CD3+, and <1% CD19+) was identified as RASFs [10]. A majority of RASFs at the first or second generation were polygonal or fusiform, with prominent nucleoli and more mitotic figures. The morphology of RASFs at the fourth generation was displayed in Figure 1, which was used in the next experiments.

### 3.2. Cell Viability Assay

**Figure 2:**
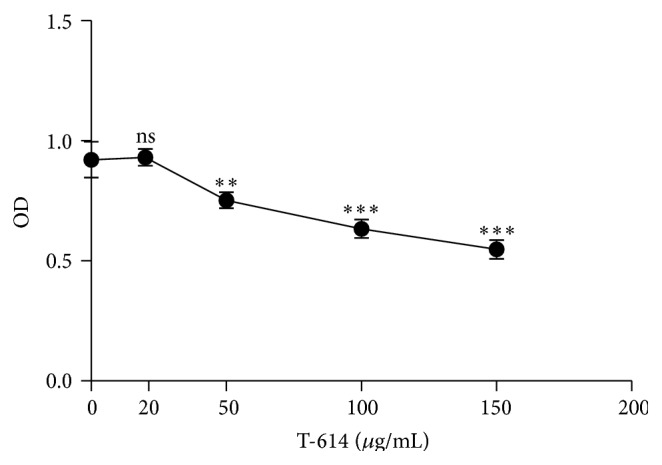
Viability of T-614 on RASFs in vitro. Cells were treated with the indicated concentrations of T-614 for 72 h. Cell viability was determined by the CCK-8 assay. Values are the mean ± SD for three independent experiments, each in triplicate. ns: not significant, ^∗∗^ *P* < 0.01, and ^∗∗∗^ *P* < 0.001 versus T-614 (0 *μ*g/mL) alone.

To test the effect of T-614 on cell viability, RASFs were treated with different concentrations of T-614 (0, 20, 50, 100, and 150 *μ*g/mL). After 72 h, CCK-8 was added to cells to assay cell viability. As shown in Figure 2, T-614 could inhibit the cell ability in a dose dependent manner; only the concentration of T-614 less than 20 *μ*g/mL did not markedly affect the growth of RASFs and was then used in current study.

### 3.3. T-614 Inhibited RANKL/OPG Expression in IL-6-Stimulated RASFs

**Figure 3:**
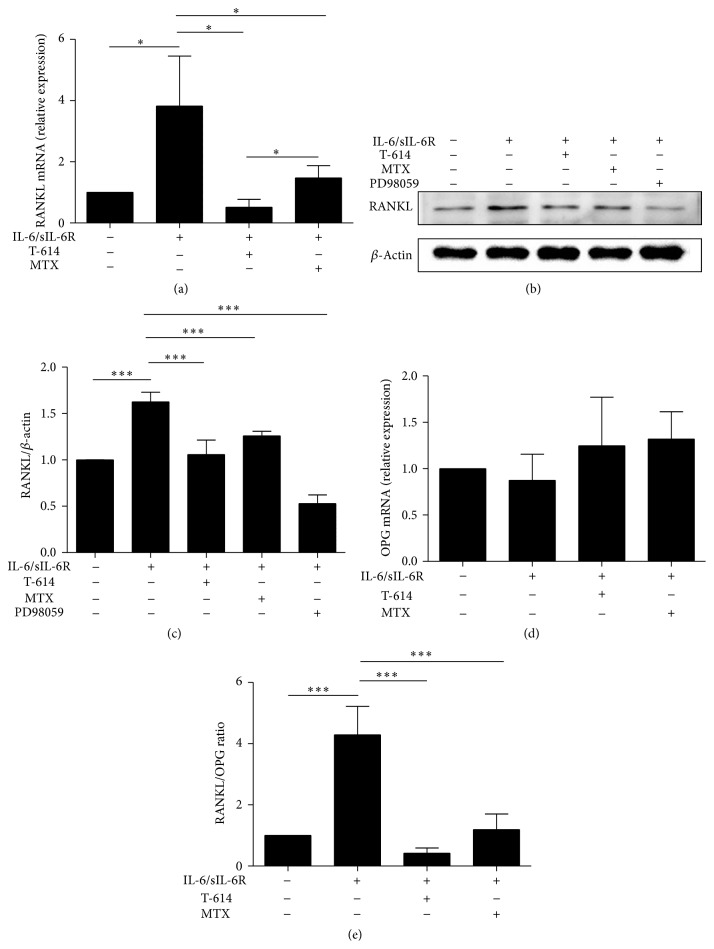
Effect of T-614 and MTX on IL-6-induced RANKL, OPG expression, and the ratio of RANKL/OPG in RASFs. RASFs were stimulated with IL-6/sIL-6R and then treated with T-614 or MTX for 72 h. The effect of T-614 and MTX on the expression of RANKL (a), OPG (d), and RANKL/OPG (e) in IL-6-induced RASFs was tested by real-time quantitative PCR analysis. Total proteins were extracted and expression levels of RANKL protein were determined by western blot (b, c). The data points shown are the mean ± SD for three independent experiments, each in triplicate. ^∗∗∗^ *P* < 0.001 and ^∗^ *P* < 0.05.

In the pathological condition of RA, RASFs in the inflamed joints could cause the exaggerated expression of multiple proinflammatory cytokines including TNF-*α*, IL-6, IL-17, and IL-1*β*, all of which resulted in an increased RANKL expression in local joint. Given that previous studies reported that only IL-1*β* or IL-6 plus sIL-6R (but not IL-6 alone) is capable of inducing RANKL expression in vitro after RASFs treatment with different cytokines [12], thus, we stimulated RASFs with IL-6/sIL-6R to induce RANKL expression in current study. As expected, a robust increased RANKL and a modest decreased OPG expression were observed in RASFs upon IL-6 stimulation (Figures 3(a), 3(b), 3(c), and 3(d)). RANKL mRNA (Figure 3(a)) and protein (Figures 3(b) and 3(c)) expression was decreased by T-614 in IL-6-induced RASFs, but OPG expression was slightly enhanced by T-614 (Figure 3(d)), which resulted in a decreased ratio of RANKL/OPG in IL-6-stimulated RASFs (Figure 3(e)). Moreover, the inhibitory role of T-614 on RANKL expression was superior to the traditional disease-modifying antirheumatic drugs (DMARDs) MTX (Figure 3(a)). PD98059, the ERK inhibitor, could also suppress RANKL protein expression (Figures 3(b) and 3(c)), indicating that ERK signal was involved in RANKL expression.

### 3.4. T-614 Inhibited the Expression of IL-17 and Its Transcription Factor RORc, as well as MMP-3 in IL-6-Stimulated RASFs

**Figure 4:**
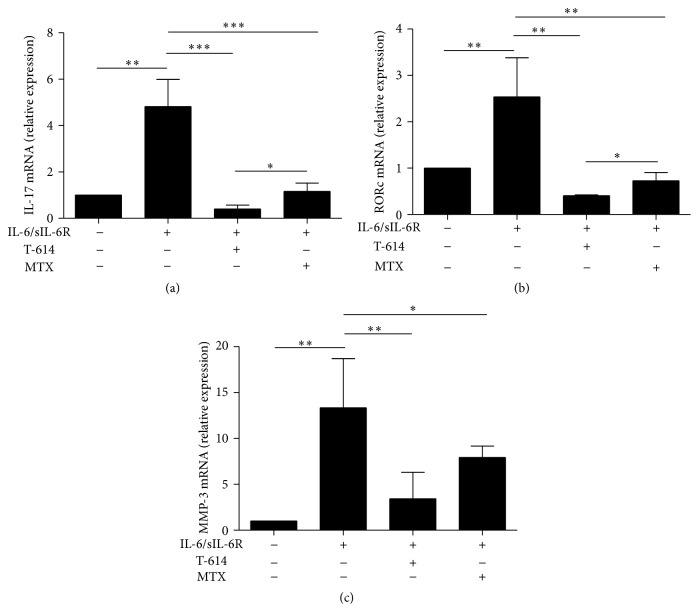
Effect of T-614 and MTX on IL-6-induced MMP-3, IL-17, and RORc mRNA expression in RASFs. RASFs were stimulated with IL-6/sIL-6R, T-614, or MTX for 72 h. The effect of T-614 and MTX on IL-6-induced IL-17 (a), RORc (b), and MMP-3 (c) mRNA expression in RASFs was tested by real-time quantitative PCR analysis. The data shown are the mean ± SD for three independent experiments, each in triplicate. ^∗∗∗^ *P* < 0.001, ^∗∗^ *P* < 0.01, and ^∗^ *P* < 0.05.

Given that previous study reported that IL-17 and MMP-3 also participated in osteoclastogenesis and played a crucial role in inflammation and bone erosion in RA patients and in CIA mice [10, 13, 14], we next evaluated potential effect of T-614 on the production of IL-17 and its transcriptional factor RORc, as well as MMP-3 mRNA expression in IL-6-induced RASFs (Figure 4). Markedly elevated expression levels of IL-17 (Figure 4(a)), RORc (Figure 4(b)), and MMP-3 (Figure 4(c)) mRNA were found in RASFs after stimulation with IL-6, which were significantly suppressed by T-614 or MTX in RASFs. Moreover, the inhibition effect of T-614 on IL-17 and RORc expression was greater than MTX (Figures 4(a) and 4(b)).

### 3.5. T-614 Inhibited RANKL Expression via ERK1/2 Signal

**Figure 5:**
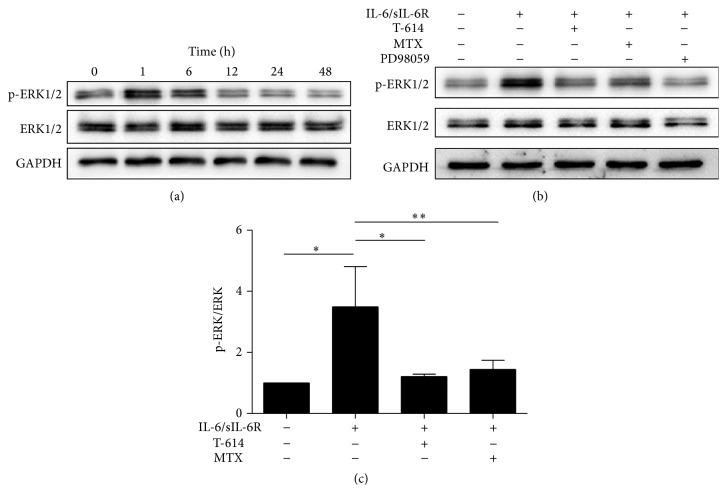
The effect of T-614 on p-ERK1/2, ERK1/2 expression in IL-6-induced RASFs. (a) RASFs were stimulated with IL-6 for 0, 1, 6, 12, 24, and 48 h; total proteins were extracted and expression levels of ERK1/2 and p-ERK1/2 protein were determined by western blot. (b, c) RASFs were cultured with PD98059 (25 *μ*M) for 1 h or not; then IL-6/sIL-6R were added, and incubation was continued for another 1 h. Total proteins were extracted and expression levels of ERK1/2 and p-ERK1/2 protein were determined by western blot. The data shown are the mean ± SD for three independent experiments, each in triplicate. ^∗∗^ *P* < 0.01 and ^∗^ *P* < 0.05.

It is reported that IL-6 exerted its action via JAK/STAT (Janus kinase/signal transducer and activator of transcription) and MAPK (mitogen-activated protein kinase) cascade by binding to its receptors [15]. Previous studies also showed that activation of ERK was necessary for RANKL expression in RASFs [5, 16, 17]. To investigate the mechanism of T-614 on suppression of RANKL expression in IL-6-stimulated RASFs, we tested the p-ERK1/2 and ERK1/2 protein expression in RASFs upon IL-6 and T-614 stimulation. The western blot suggested that IL-6 could induce ERK1/2 phosphorylation in RASFs. The peak of ERK1/2 phosphorylation was at 1 h after the addition of IL-6 into cells (Figure 5(a)). T-614 and MTX could markedly decrease the phosphorylation of p-ERK1/2 (*P* < 0.05; Figures 5(b) and 5(c)), indicating that the inhibitory role of T-614 on RANKL protein expression might be through ERK1/2 pathway.

## 4. Discussions

The novel disease-modifying antirheumatic drug, T-614, has a reported effect on preventing the inflammatory and destructive processes of RA by inhibiting the production of immunoglobulins and various inflammatory cytokines (IL-1, IL-6, IL-8, and TNF-*α*), exerting a unique mechanism for RA therapy [18]. Here, we confirmed the anti-inflammatory role of T-614; moreover, our data suggested that T-614 could downregulate the ratio of RANKL/OPG via blocking ERK activation, suggesting a novel mechanism of T-614 on inhibition of bone destruction in RA.

Osteoclast is the cell ultimately responsible for destruction in RA. A mass of evidences indicates that RANKL plays an important role in regulating osteoclast development [19]. RANKL is known to be produced by a number of different cell types including T cells, B cells, dendritic cells, macrophages, and synovial fibroblasts in RA [20–22]. OPG acts as its natural decoy receptor by blocking the RANK/RANKL interaction and the elevated ratio of RANKL/OPG may represent a high state of osteoclastogenesis and high activity of bone degradation. Given that RASFs were one of the major cells that express RANKL [23], here, RASFs were stimulated with IL-6 to induce RANKL expression. We found, for the first time, that T-614 has a potent ability to decrease the ratio of RANKL/OPG, which suggested that the therapeutic effect of T-614 on preventing disease progression, at least in part, attributed to its regulation role on RANKL/OPG axis hence contributing to suppress osteoclastogenesis [24].

IL-17, also produced by RASFs [7], plays a crucial role in inflammation and bone erosion in RA patients and in CIA mice [12, 13]. IL-17 could also upregulate the expression of RANKL in osteoblasts and synovial fibroblasts and then amplify the bone destruction in collagen-induced arthritis (CIA) mice. RORc is the transcription factor for IL-17. Consistent with recent report that T-614 could suppress IL-17 signal in RASFs [5], our data proved that T-614 could significantly downregulate the production of IL-17 and RORc in IL-6-induced RASFs. Moreover, T-614 showed a greater inhibitory effect on IL-17 and RORc expression than MTX.

MMP-3 has been suggested to play a pivotal role in the cartilage destruction in RA. Patients with joint injury have been found to have persistently increased proMMP-1 and proMMP-3 levels in synovial fluid, which mainly came from RASFs [25]. Serum MMP3 was correlated with IL-8, IL-6, IFN-*γ*, CRP and cartilage breakdown in 128 patients [26] and have been suggested to be a good laboratory index to evaluate the joint injury status and therapeutic effect [27]. In our study, IL-6 increased the production of MMP-3 in RASFs, confirming that inflammatory environment of joints was the major cause of high expression of MMP-3. Here, our data demonstrated that T-614 could decrease MMP-3 production in IL-6-induced RASFs, implying that T-614 could reduce disease activity and bone erosion of RA.

Substantial evidence has suggested that ERK phosphorylation was involved in RANKL [5, 16, 17], IL-17 [28], and MMP-3 [29] expression in RASFs. In order to investigate the potential pathway of T-614 on RAKL/OPG expression in RASFs, we studied the effect of T-614 on ERK1/2 signaling. Our data showed that the phosphorylation of ERK1/2 was triggered after the stimulation of RASFs by IL-6 and reached peak at 1 h after stimulation with IL-6. The level of ERK1/2 phosphorylation could be abolished by T-614 and MTX. The high expression of RANKL protein in IL-6-induced RASFs could be cancelled by ERK inhibitor, PD98059, confirming that T-614 inhibited RANKL expression via ERK1/2 signal. Taken together, our data suggested that T-614 could inhibit RANKL expression in RASFs via ERK1/2 pathway.

## 5. Conclusions

T-614 has been confirmed as a highly effective drug for RA therapy and has been widely used in clinics in China and Japan. The present study demonstrates, for the first time, that T-614 could decrease the RANKL expression and downregulate the ratio of RANKL/OPG in RASFs via blocking ERK1/2 phosphorylation; we also confirmed that T-614 could suppress IL-17 and MMP-3 expression in IL-6-induced RASFs. This study also identified that the immunosuppressive effects in RANKL and IL-17 expression of T-614 on RASFs were stronger than MTX; our data provide a novel insight into the mechanisms of antiarthritic effect in T-614.

## Abbreviations

RANK: Receptor activator of NF-*κ*B
RANKL: Receptor activator of the NF-*κ*B ligand
OPG: Osteoprotegerin
RA: Rheumatoid arthritis
RASFs: Rheumatoid arthritis synovial fibroblasts
IL-17: Interleukin-17
RORc: Retinoid-related orphan receptor C
MMP-3: Matrix metalloproteinase-3
MTX: Methotrexate
TNF: Tumor necrosis factor
CIA: Collagen-induced arthritis
sIL-6R: Soluble IL-6 receptor.
